# Expression profile analysis of prognostic long non-coding RNA in adult acute myeloid leukemia by weighted gene co-expression network analysis (WGCNA)

**DOI:** 10.7150/jca.31234

**Published:** 2019-08-19

**Authors:** Cun-Te Chen, Pei-Pei Wang, Wen-Jian Mo, Yu-Ping Zhang, Wei Zhou, Ting-Fen Deng, Ming Zhou, Xiao-Wei Chen, Shun-Qing Wang, Cai-Xia Wang

**Affiliations:** 1 Department of Hematology, Guangzhou First People's Hospital, School of Medicine, South China University of Technology, Guangzhou, Guangdong, China;; 2 Department of Oncology, Guangzhou First People's Hospital, School of Medicine, South China University of Technology, Guangzhou, Guangdong, China.

**Keywords:** acute myeloid leukemia, lncRNA, WGCNA, prognosis, risk stratification

## Abstract

**Background:** Long non-coding RNAs (lncRNAs), which are over 200 nt in length, have a key role in tumorigenesis and disease progression. To explore the role of prognostic lncRNAs in adult acute myeloid leukemia (AML), the expression profiles of lncRNAs and mRNAs in AML were analyzed.

**Methods:** The RNAseq data of 167 adult AML patients and the corresponding clinical information were downloaded from The Cancer Genome Atlas (TCGA), which is a publicly available database. The RPKM values of the RNAseq data were subjected to weighted gene co-expression network analysis (WGCNA) in modularization.

**Results:** We identified survival specific lncRNAs and mRNAs, which were divided into modules by coexpression analysis. The lncRNAs were mainly annotated into “Fc gamma R-mediated phagocytosis”. The hub lncRNA and co-expressed mRNAs were further selected for analysis of risk stratification. LncRNA-LOC646762 may contribute to AML through the "endocytosis" signaling pathway. Finally, the expression levels of LOC646762 and co-expressed CCND3, CBR1, C10orf54, CD97 and BLOC1S1 in the adult AML patients and healthy volunteers were validated by qRT-PCR, and then their roles in prognosis and risk stratification were identified.

**Conclusions:** Prognostic lncRNA-LOC646762, which may contribute to AML through the "endocytosis" signaling pathway, may act as a biomarker for predicting the survival of adult AML patients, as well as for risk stratification.

## Introduction

Acute myeloid leukemia (AML) is a heterogeneous hematological malignancy that threatens human health 1. It is the most common histological type of acute leukemia in adults, with an incidence of 3.7 per 100,000 individuals, and the disease progresses rapidly, with an age-dependent mortality of 2.7 to nearly 18 per 100,000 individuals 2. The rapid advances in high-throughput technologies, such as microarray and next-generation sequencing, have provided increasingly precise prediction for the diagnosis and prognosis of AML patients, which enables more efficient personal treatment for patients 3. However, most of the transcripts detected by high-throughput sequencing are non-coding genes and are likely to be involved in long noncoding RNAs (lncRNAs), which are over 200 nt in length. As researchers have conducted extensive research on the pathogenesis of AML, it has been established that abnormal lncRNAs may play an important role in the cancer pathways 4, 5. Furthermore, lncRNAs could be biomarkers of acute leukemia subtypes, and therefore could be used for categorization and risk stratification of leukemia 6, 7.

Bioinformatics analysis is a method that is increasingly used for the exploration of target genes and proteins. Weighted gene co-expression network analysis (WGCNA) is a systematic biological method used to describe correlation patterns among genes in microarray samples, which can identify clusters (modules) of highly correlated genes for the investigation of potential functions 8. Recent studies demonstrate that WGCNA has been widely applied in screening and identification of susceptibility genes and candidate targets for disease 9, 10.

The Cancer Genome Atlas (TCGA), a collaboration between the National Cancer Institute (NCI) and National Human Genome Research Institute (NHGRI), has provided comprehensive, multi-dimensional maps of the key genomic changes in 33 types of cancer, including AML 11. TCGA is a publicly available database that contains 200 sets of microarray data on AML samples and the corresponding clinical information, which has been widely used by researchers 12, 13.

In the present study, the RNAseq data of adult AML in the TCGA was used for conducting WGCNA in order to analyze the prognostic genes, with an emphasis on the role of prognostic lncRNAs in the modularization process and risk stratification. Specifically, we validated the expression levels of the lncRNA and co-expressed mRNAs in adult AML patients and healthy volunteers using quantitative real-time PCR (qRT-PCR), and identified the prognostic and risk stratification roles of the lncRNA and co-expressed mRNAs.

## Materials and Methods

### Patients' samples

Bone marrow samples from 70 patients who were newly diagnosed with AML were collected at our institution between March 2015 and September 2018. AML was diagnosed according to the World Health Organization MICM (morphology, immunology, cytogenetics, and molecular biology) classification criteria. The inclusion criteria was as follows: (1) Blasts ≥20% of the bone marrow nucleated cells (ANC); or (2) blasts <20% of bone marrow nucleated cells (ANC), but with t(15,17), t(8,21) or inv(16)/t(16,16). The exclusion criteria were as follows: (1) Age <18 years; or (2) no complete follow-up information. There were 31 male patients and 39 female patients, with an age range from 20 to 85 years old. Histologically, 3 patients had M1, 17 patients had M2, 11 patients had M3, 5 patients had M4, 32 patients had M5, and 2 patients had M6. The bone marrow samples were collected in heparinized tubes before treatment and shipped to the laboratory within 24 to 36 hours. The leukemic cells were isolated by density gradient centrifugation using 1.077 g/mL Ficoll-Isopaque (Pharmacia). The proportion of leukemic cells was estimated using May-Grünwald-Giemsa-stained cytocentrifugate preparations and light microscopy. The cell samples selected for analysis contained at least 90% blasts after separation. Pellets of 2 to 10 million cells were stored in TRIzol (Invitrogen, Carlsbad, CA, USA) and immediately frozen at -80˚C. Clinical follow-up data were retrieved with the last follow-up on September 30^th^, 2018. The median follow-up time for the surviving patients was 693 days (range, 20 - 1291 days) (Table S1). Bone marrow samples from ten anonymized healthy volunteers were included as control samples.

### TCGA data processing

**Figure 1:**
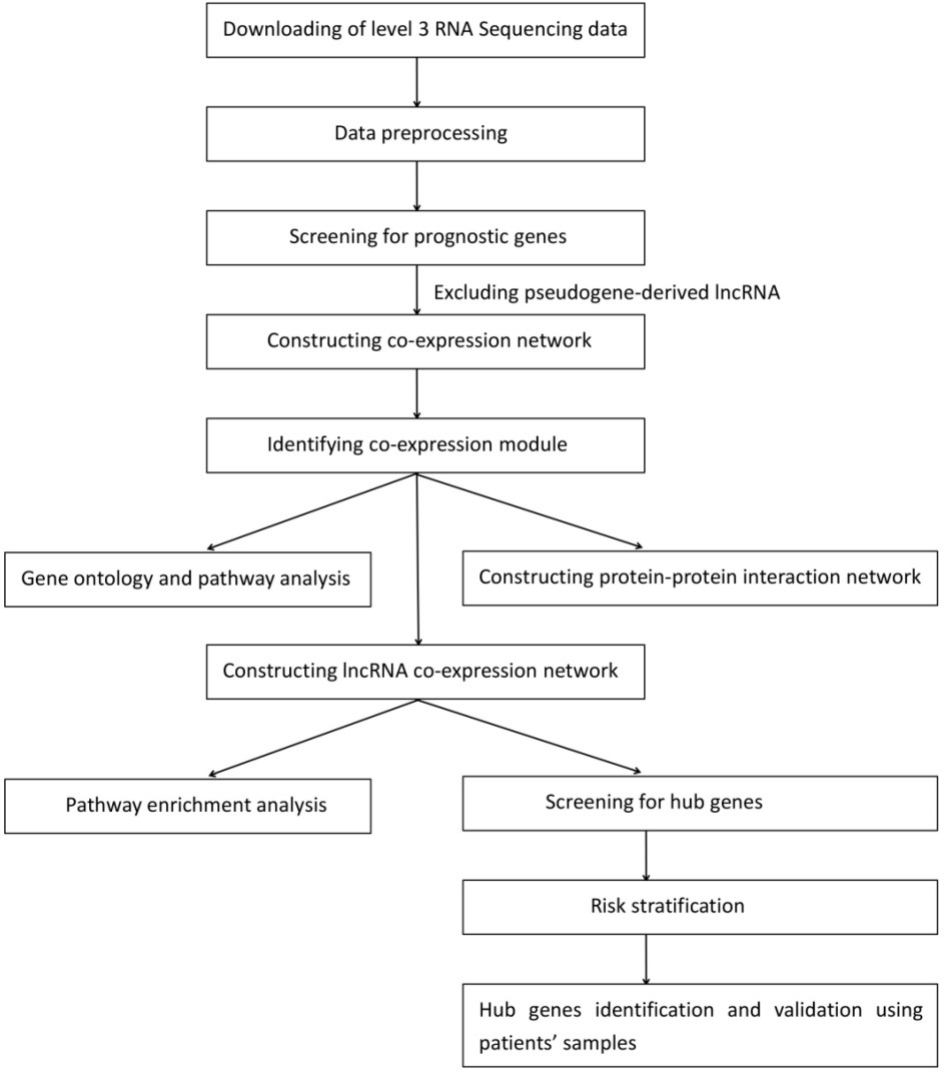
A workflow of the data downloading, processing, analysis and validation.

A workflow of the study is presented in Fig. 1. The level 3 RNA sequencing data and the corresponding clinical information of the adult AML patients, which consisted of 179 patients and 20442 genes, were obtained from TCGA database (https://cancergenome.nih.gov/) using the R package RTCGAToolbox 14. The RPKM values were used for the subsequent analysis. The exclusion criteria were as follows: (I) Patient was alive and the number of days to the last follow-up was unavailable; or (II) patient was deceased and the number of days until they succumbed to the disease was unknown. After the exclusion of patients based on the aforementioned criteria, 167 AML patients were identified for univariate Cox regression analysis using the coxph function in R (version 3.5.1, https://www.r-project.org/). The exclusion criteria of the genes were set as follows: (I)* P*-value ≥0.05; or (II) pseudogene-derived lncRNA. The selected mRNAs and lncRNAs were significantly associated with overall survival. Subsequently, 167 adult AML patients and 3619 genes, which contained 3535 mRNAs and 84 lncRNAs, were selected for WGCNA (Table S1). The clinical characteristics of testing and validation cohort were showed in Table 1. The RNA sequencing data and the corresponding clinical information of the adult AML patients are publicly available in TCGA, so the approval of the local ethics committee was not required.

### Weighted gene co-expression network analysis (WGCNA)

The WGCNA R package was used to construct a weighted correlation network between the prognostic lncRNA and mRNAs 8. In the network, we used the pairwise Pearson coefficient to evaluate the weighted co-expression relationship between all genes in the adjacency matrix. The soft threshold was used to ensure a scale-free network. In the unsigned co-expression network, genes with high absolute correlations were clustered into the same module. The modules were also identified by hierarchical clustering of the weighted coefficient adjacency matrix to calculate the topological overlap matrix (TOM) 15. In addition, the topological overlap of intramodules and adjacency modules was used for selecting the functional modules.

### Pathway analysis and PPI network establishment

The lncRNA co-expressed mRNAs, which were calculated by WGCNA, underwent pathway analysis and protein-protein interaction (PPI) network establishment. The Database for Annotation, Visualization and Integrated Discovery (DAVID, https://david.ncifcrf.gov/) tool was used for Kyoto Encyclopedia of Genes and Genomes (KEGG) pathway analysis. The PPI network of the lncRNA co-expressed mRNAs was established using the Search Tool for the Retrieval of Interacting Genes/Proteins (STRING, https://string-db.org/). In the network, nodes represent genes and edges represent the interactions between the nodes. The highly important nodes in the network, the hub nodes, were obtained according to the k-core.

### X-tile for the optimal cut-points

X-tile (version 3.6.1, Yale University, New Haven, CT, USA) 16 is a new bioinformatics tool that is used to determine the optimal cut-point for risk stratification. The X-tile plots were built by dividing the marker data into the following three groups: Low, middle and high. All possible cut-points of the marker data were assessed by the log-rank test for survival analysis. The X-tile can determine the optimal cut-points of the marker data by selecting the highest χ^2^ value and lowest P-value.

### Quantitative real-time PCR (qRT-PCR)

For the reverse transcription PCR, a Reverse Transcription Kit (Promega, Madison, Wisconsin, USA) was used to reverse transcribe the total RNA to cDNA according to the manufacturer's protocol. The expression levels of the genes were quantified using SYBR Green Master Mix (Promega, Madison, Wisconsin, USA), and 18S rRNA was used as an internal control. The primers for qRT-PCR are listed in Table 2. qRT-PCR was performed on ViiA^TM^ 7 System software (Thermo Fisher Scientific, Waltham, MA, USA). The results were normalized to the expression of 18S rRNA and are presented as the fold change (2^-ΔΔCT^).

### Statistical analysis

All statistical analyses were conducted using SPSS 16.0 software (SPSS Inc, Chicago, IL, USA) and GraphPad Prism 5.0 software (GraphPad, CA, USA) as appropriate. The overall survival rates were calculated using the Kaplan-Meier method, and the log-rank test was used for comparisons. A time-dependent ROC curve was created to evaluate the predicted ability of genes 17. Scatter plots were produced to visualize the correlationship between the lncRNA and mRNAs. The survival data were evaluated using a multivariate Cox proportional hazards regression model. A *P*-value of <0.05 was considered significant.

## Results

### Construction of co-expression modules

**Figure 2:**
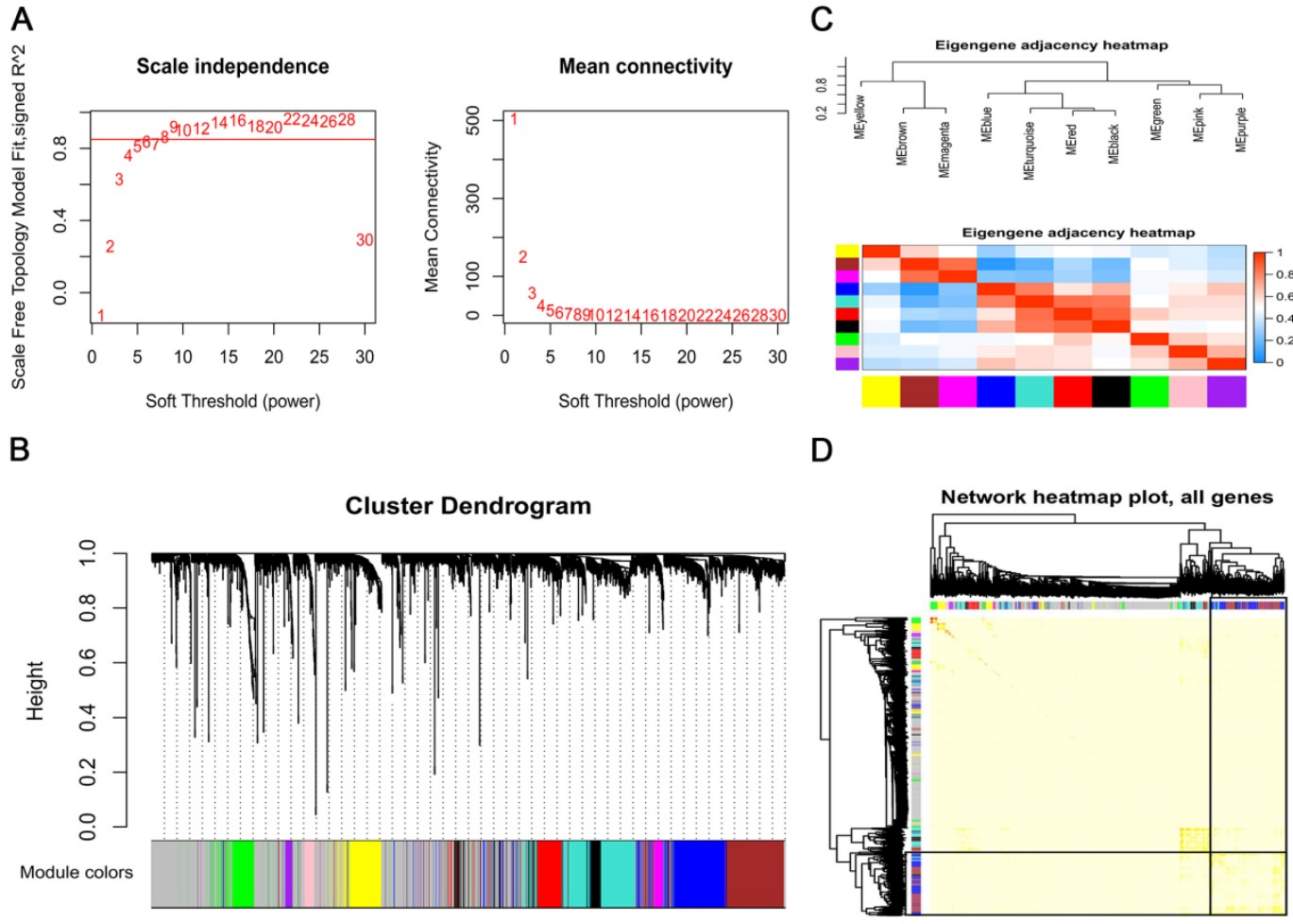
** Construction of a weighted gene co-expression network.** (A) Analysis of the scale-free topology model fit index for soft threshold powers (β) and the mean connectivity for soft threshold powers. (B) A cluster dendrogram was built based on the dissimilarity of the topological overlap, which presents 11 gene co-expression modules in AML. The grey module indicates none co-expression between the genes. (C) Correlated heatmap plot of the adjacency modules in the WGCNA. The rectangle of each row and column represents a module eigengene. In the correlated heatmap plot, light blue represents low adjacency, while red represents high adjacency. (D) Topological overlap matrix (TOM) plot in the gene co-expression network of the intramodules. In the TOM plot, the light color indicates topological overlap, while the darker color indicates high topological overlap. The gene dendrogram and corresponding modules are shown along the left and top of the TOM plot. The intersection of the two rectangles indicates the topological overlap in the blue module.

To explore the co-expression patterns of the survival-specific lncRNA and mRNAs in AML, gene co-expression network analysis (WGCNA) was performed. A total of 3619 survival-specific genes in TCGA, which consisted of 84 lncRNA and 3535 mRNAs, were included into the WGCNA. The power of β = 8 (scale free R^2^ = 0.85) was set as the soft threshold for a scale-free network (Fig. 2A). As shown in Fig. 2B, the cluster dendrogram contained eleven co-expression modules, which are represented by black, blue, brown, green, grey, magenta, pink, purple, red, turquoise and yellow. The number of genes in each of these modules was 122, 327, 312, 139, 1046, 47, 49, 37, 135, 327 and 173, respectively. The co-expressed genes were primarily clustered in the blue and turquoise modules. In order to explore the function of a module, we randomly selected the blue module for further analysis. By generating the eigengene adjacency heatmap, we revealed that the blue module exhibited a strong correlation with the other modules, indicating that the blue module was a central module in all of the modules (Fig. 2C). We also constructed a TOM plot and found that the genes in the blue module have strong co-expression relationships (Fig. 2D).

### Enrichment analysis and PPI network of the genes in the blue module

**Figure 3:**
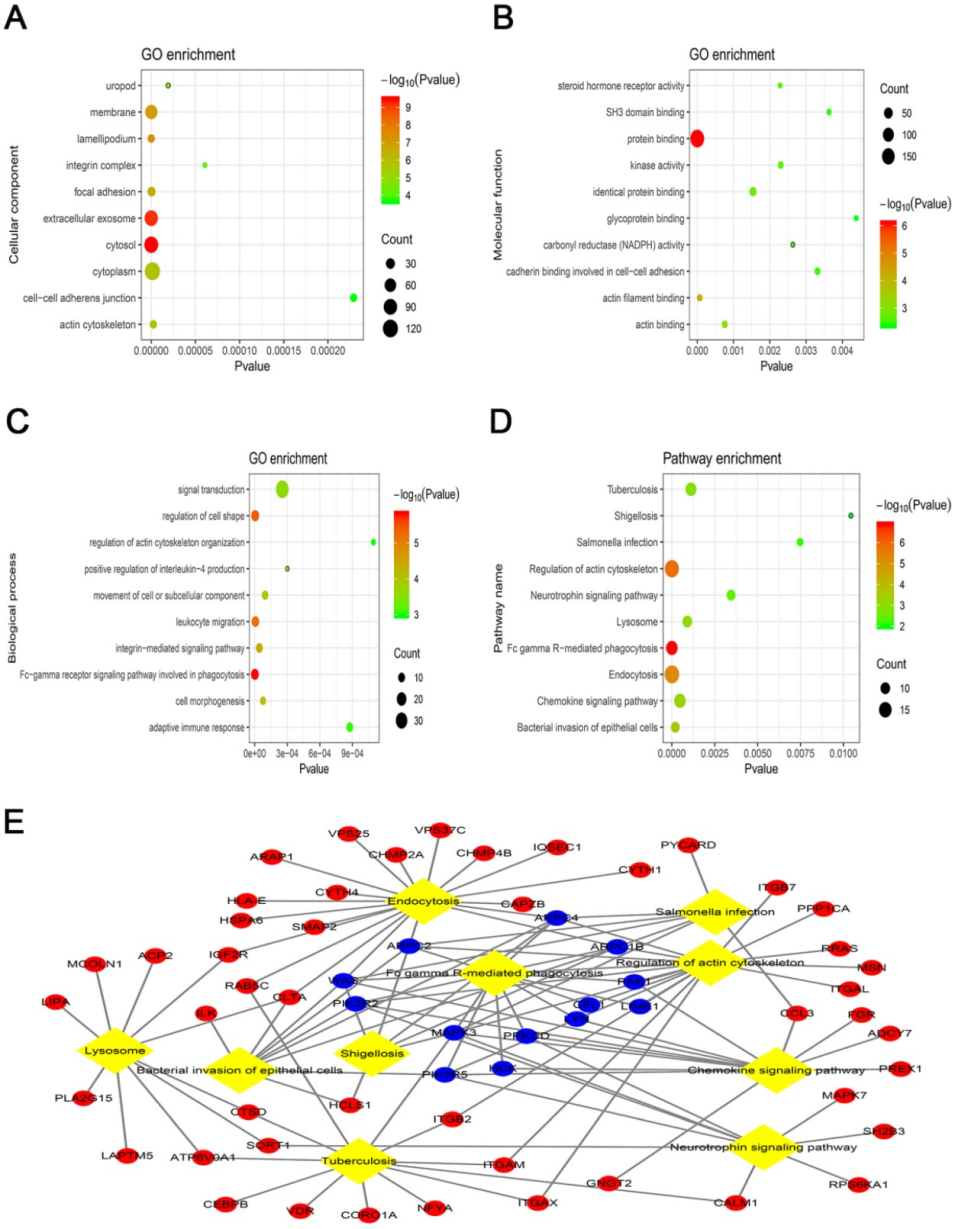
Top 10 enrichment of the gene ontology (GO) terms and Kyoto Encyclopedia of Genes and Genomes (KEGG) pathways of the mRNAs in the blue module. (A) Cellular component. (B) Molecular function. (C) Biological process. (D) KEGG pathways analysis. (E) Interaction and overlapping of the top 10 pathways. The diamond represents the pathway, while the circle indicates the mRNA in the pathway. The blue circle represents the mRNA in the Fc gamma R-mediated phagocytosis pathway.

To clarify the biological functions associated with OS in the blue module, the co-expressed mRNA were annotated with GO and KEGG. The GO analysis revealed that the most significant GO terms were “cytosol” (ontology: cellular component), “protein binding” (ontology: molecular function) and “Fc-gamma receptor signaling pathway involved in phagocytosis” (ontology: biological process) (Fig. 3A-C). Furthermore, the KEGG pathway analysis showed that the most significantly enriched pathway associated with overall survival (OS) was “Fc gamma R-mediated phagocytosis” (Fig. 3D). Among the KEGG pathways, the “Fc gamma R-mediated phagocytosis” and “regulation of actin cytoskeleton” pathways were considered to be the most central functions because the exchanges with other pathways strongly depended on their existence (Fig. 3E).

**Figure 4:**
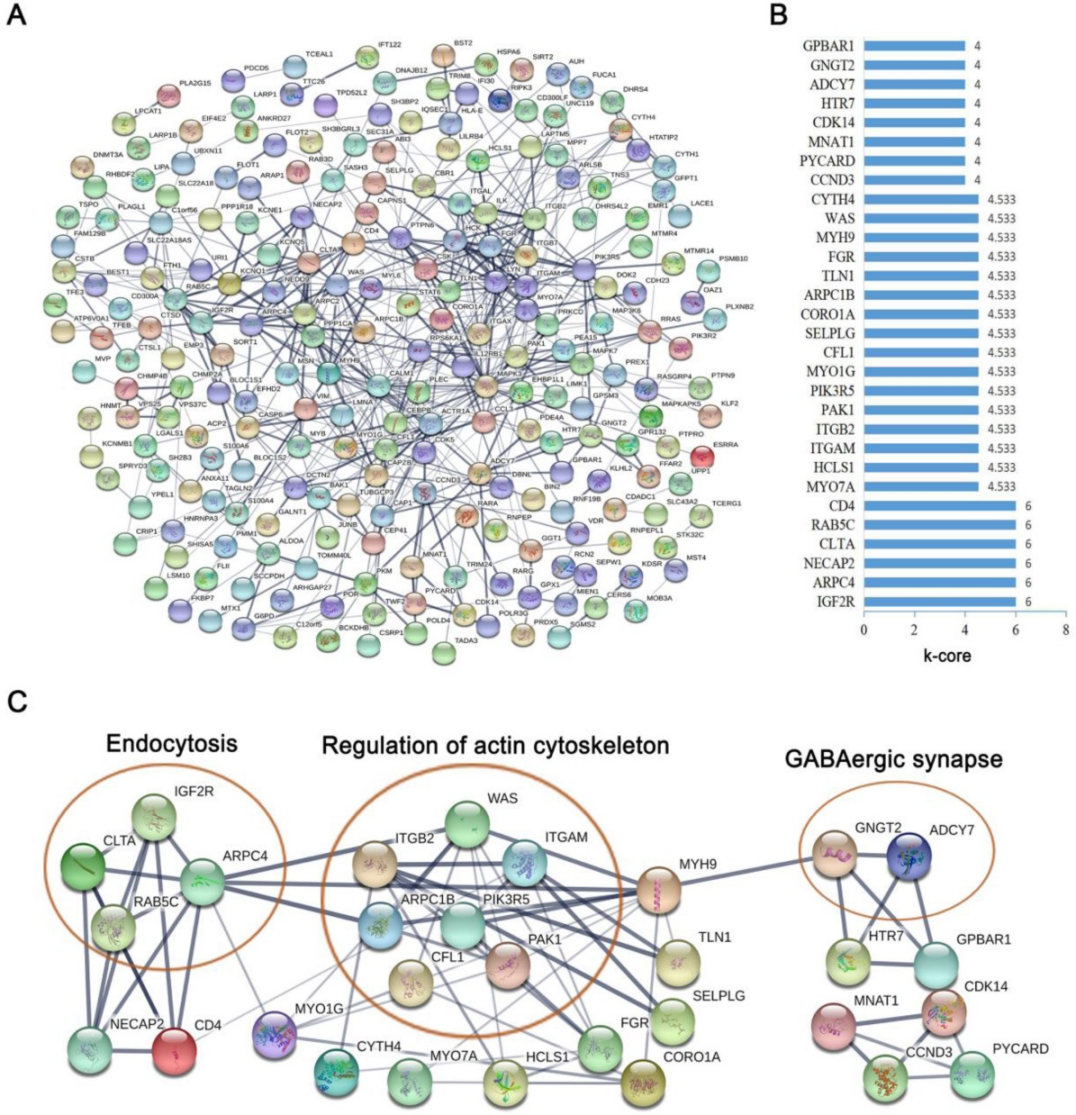
** Protein-protein interaction network by STRING software.** (A) The protein-protein interaction network of the co-expressed mRNAs in the blue module was constructed, which was based on the confidence score of the experimental and computational interaction. (B) The thirty top k-core genes in the network. (C) The thirty top k-core genes involved in the four core subnetworks that enriched three pathways. The ellipses represent the signaling pathway.

To further investigate the function of the co-expressed genes in the blue module at the protein level, the STRING database was used to screen for functional genes, which provided a visual annotation network to identify the structural and functional properties of the proteins. The PPI network consisted of 231 nodes and 579 edges (Fig. 4A). The k-core was used to determine the core genes of the PPI network, and the 30 highest k-core genes contained four core subnetworks (Fig. 4B). The four core subnetworks were enriched in three pathways, which included “endocytosis”, “regulation of actin cytoskeleton” and “GABAergic synapse” (Fig. 4C).

### LncRNA co-expression network establishment and pathway analysis

**Figure 5:**
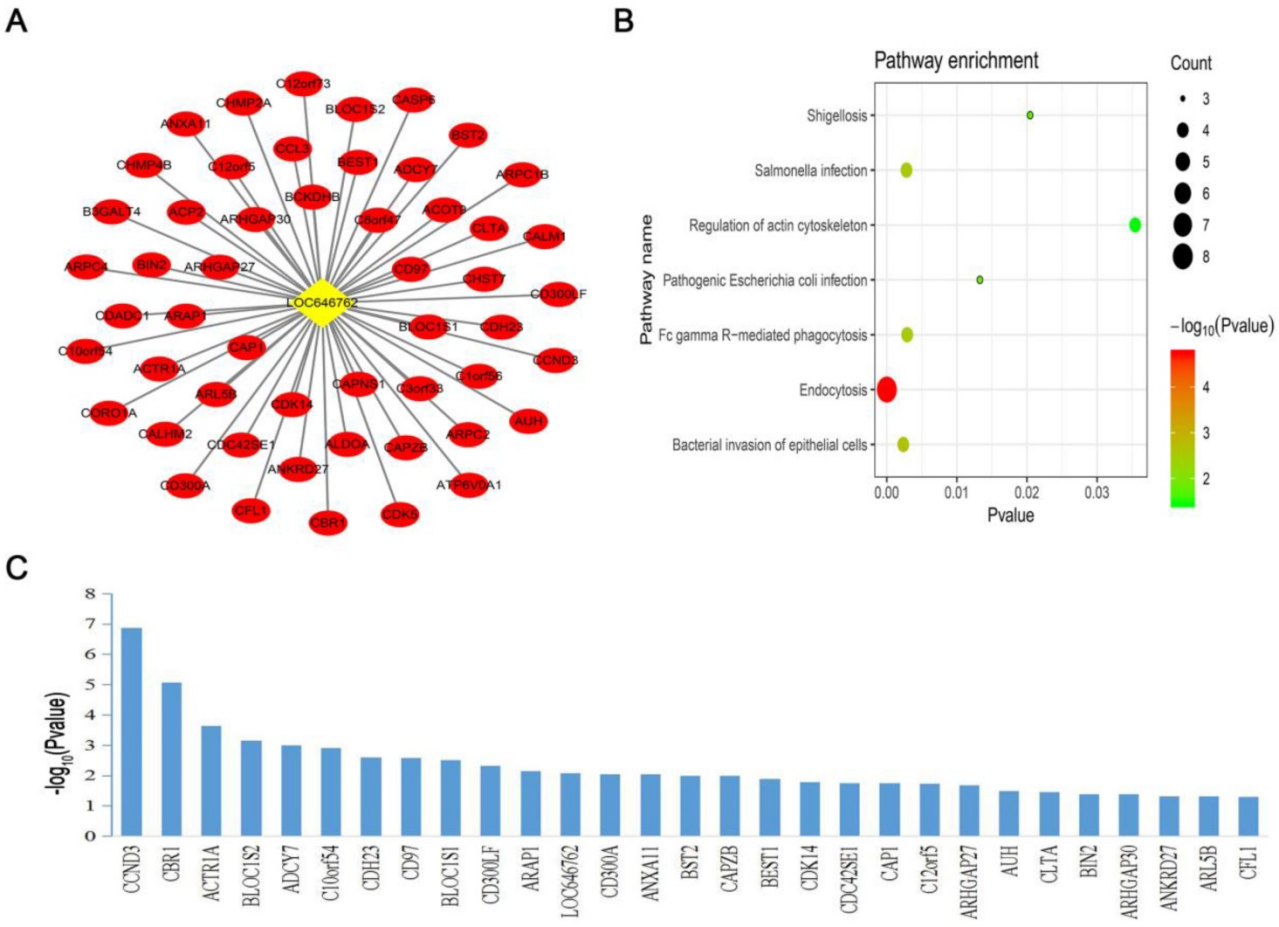
** Construction of the lncRNA co-expression network and pathway analysis.** (A) A co-expression network of lncRNA-LOC646762 and 52 mRNAs was built in the blue module. (B) Pathway analysis of the co-expressed genes of LOC646762. (C) The prognostic mRNAs of the LOC646762 co-expression network after screening with a log-rank test. A *P*-value of <0.05 was considered significant.

To further explore the co-expression pattern and function of the survival-specific lncRNA and 326 mRNAs in the blue module, we constructed a co-expression network of lncRNA-LOC646762 and performed KEGG pathway analysis. The co-expression network of LOC646762 was composed of 52 mRNAs (Fig. 5A). Moreover, the KEGG pathway analysis revealed that LOC646762 was mainly involved in seven important pathways. Among these pathways, the most significant pathway was “endocytosis” (Fig. 5B).

### Prognostic lncRNA and mRNAs in the blue module

To determine the prognostic genes of the LOC646762 co-expression network, we selected 29 genes using the log-rank test (*P*<0.05), which included LOC646762 and 28 mRNAs (Fig. 5C). Furthermore, five hub genes containing LOC646762, CCND3, CBR1, C10orf54, CD97 and BLOC1S1, were obtained from the 29 prognostic genes for risk stratification in adult AML using multivariate Cox regression analysis (Table 3).

**Figure 6:**
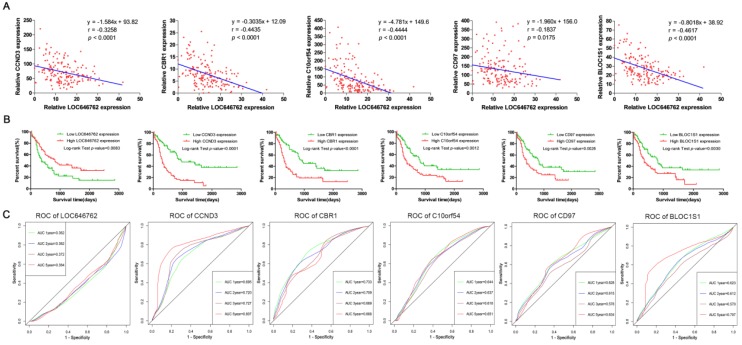
** Prognostic lncRNA and mRNAs in adult AML.** (A) The correlation between the expression of LOC646762 and the prognostic mRNAs in AML. r: Pearson correlation coefficient. (B) Kaplan-Meier curves for overall survival (OS) in low and high expression of the prognostic lncRNA and mRNAs. (C) Time-dependent ROC curves for the prognostic lncRNA and mRNAs. Green, blue, brown and red indicate the sensitivity curve, and grey represents the identify line.

The relationship between the lncRNA and mRNAs was further elucidated, and Fig. 6A demonstrates that the expression of LOC646762 was negatively correlated with the expression of CCND3, CBR1, C10orf54, CD97 and BLOC1S1 (r<0). To provide more prognostic information for the clinic, we evaluated the prediction ability of the hub genes in AML patients. As shown in Fig. 6B, low expression of LOC646762 predicted a poor prognosis, while high expression of CCND3, CBR1, C10orf54, CD97 and BLOC1S1 indicated adverse outcomes in AML patients. The results were in line with the time-dependent ROC curves. LOC646762 had an inverse relationship with the prognosis (AUC<0.5), while CCND3, CBR1, C10orf54, CD97 and BLOC1S1 were positively correlated with prognosis (AUC>0.5) (Fig. 6C).

### Risk stratification in adult AML patients

**Figure 7:**
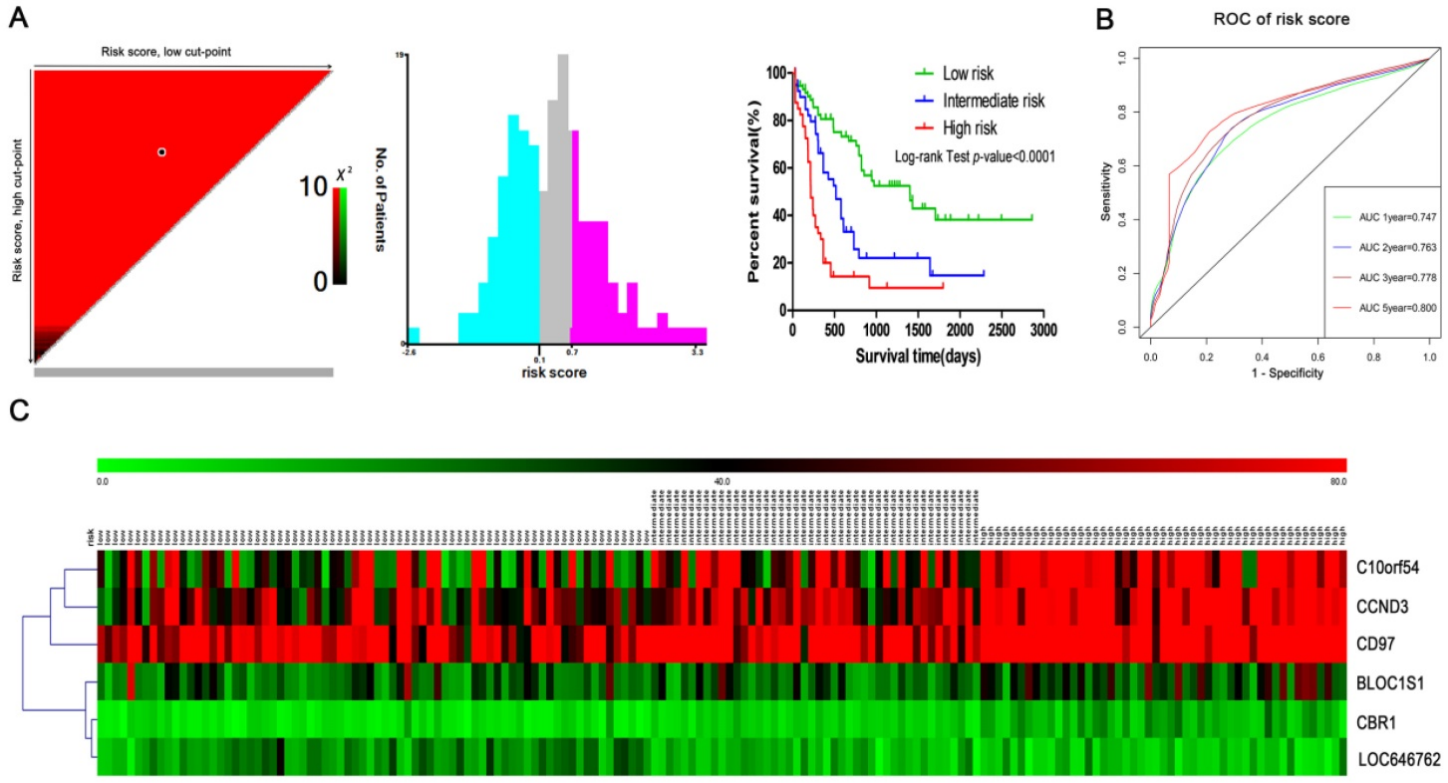
** Risk stratification in AML.** (A) The plot shows the log-rank χ^2^ values produced by the two optimal cut-points, which divides the risk score into high, middle and low subsets. The red color of the cut-points is inversely related to survival, while the green color indicates a positive correlation. The optimal cut-point appears at the brightest pixel (red or green). (B) Time-dependent ROC curves for the risk score. Green, blue, brown and red indicate the sensitivity curve, and grey represents the identify line. (C) Heatmap for the lncRNA and mRNA expression levels among the three risk groups in all 167 adult AML patients.

A prognostic model was constructed based on the weight on OS of each of the genes in the multivariate Cox regression analysis. The risk score = (-0.06 x RPKM value of LOC646762) + (0.011 x RPKM value of CCND3) + (0.12 x RPKM value of CBR1) + (-0.005 x RPKM value of C10orf54) + (0.005 x RPKM value of CD97) + (-0.03 x RPKM value of BLOC1S1). Subsequently, the patients were divided into three groups (low risk, intermediate risk and high risk) using the X-tile tool to determine the optimal cut-points of the risk scores (Fig. 7A). The Kaplan-Meier curve for OS among the low risk, intermediate risk and high risk groups demonstrated that it was significantly different (*P*<0.0001). At the same time, the AUC for 1-year, 2-year, 3-year and 5-year was 0.747, 0.763, 0.778 and 0.800, respectively (Fig. 7B). In addition, the heatmap plot indicated that the expression of the six genes was significantly different among the low risk, intermediate risk and high risk groups, and the patients associated with an adverse prognosis were mainly classified into the intermediate and high risk groups (Fig. 7C).

### Validation of the prognostic lncRNA and mRNAs in the clinical samples of adult AML

**Figure 8:**
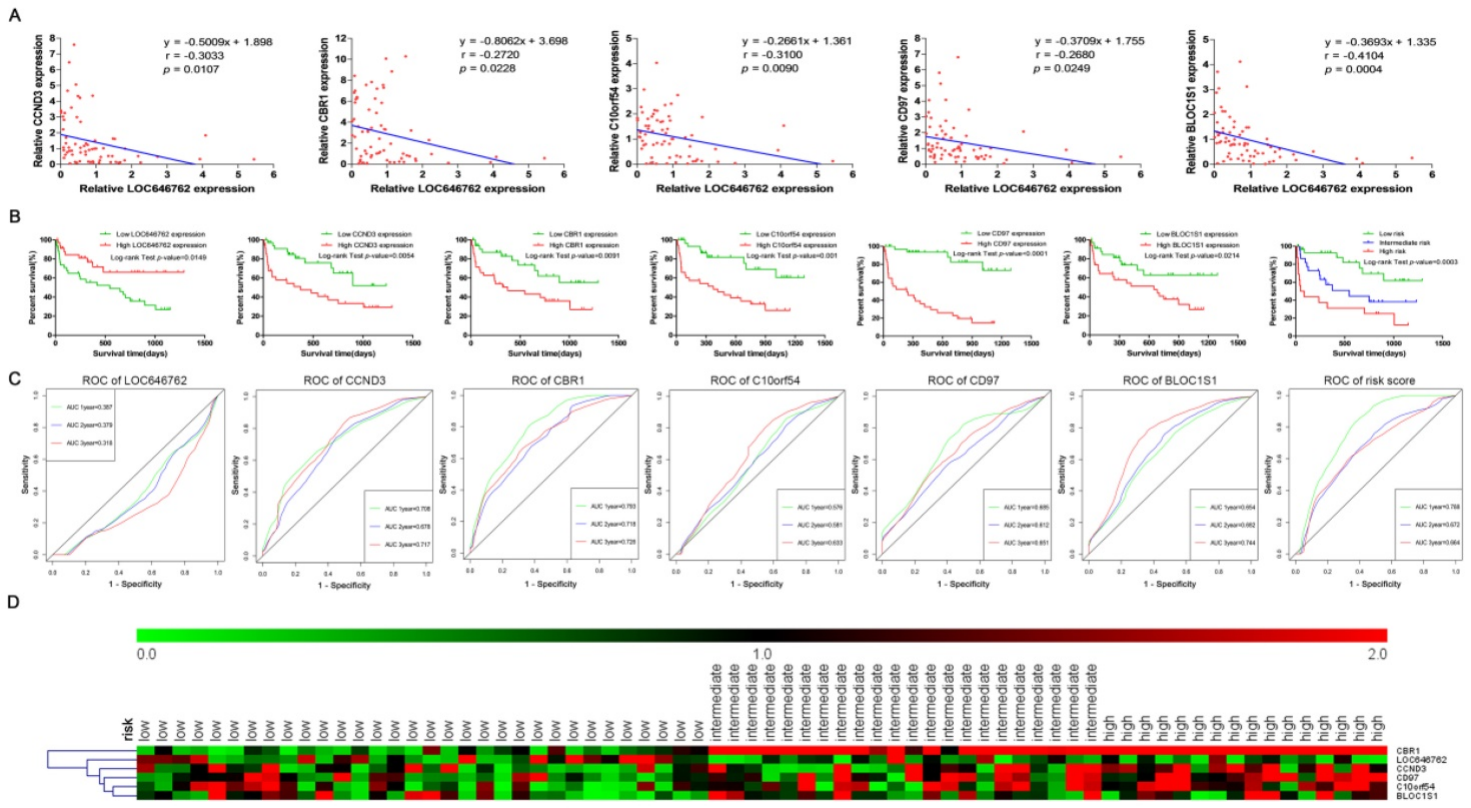
** Validation of LOC646762, CCND3, CBR1, C10orf54, CD97 and BLOC1S1 based on the clinical samples of adult AML.** (A) The correlation between the expression of LOC646762 and the mRNAs in AML. (B) Kaplan-Meier curves for the lncRNA, mRNAs and risk stratification. (C) Time-dependent ROC curves for the lncRNA, mRNAs and risk score. Green, blue, brown and red indicate the sensitivity curve, and grey represents the identify line. (D) Heatmap for the lncRNA and mRNA expression levels among the low, intermediate, and high risk groups in 70 AML patients.

The expression of LOC646762 was negatively correlated with the expression of CCND3, CBR1, C10orf54, CD97 and BLOC1S1 (*P*<0.05, Fig. 8A). As shown in Fig. 8B and C, the Kaplan-Meier curves and time-dependent ROC curves suggested that high expression of LOC646762 was associated with a favorable prognosis, while high expression of CCND3, CBR1, C10orf54, CD97 and BLOC1S1 predicted a poor prognosis. The risk score = (-0.06 x relative expression of LOC646762) + (0.011 x relative expression of CCND3) + (0.12 x relative expression of CBR1) + (-0.005 x relative expression of C10orf54) + (0.005 x relative expression of CD97) + (-0.03 x relative expression of BLOC1S1). Based on the optimal cut-points 0.1 and 0.7 of the risk score, the AML patients were divided into low risk, intermediate risk and high risk groups. The survival analysis demonstrated that there was a significant difference (*P*<0.05). Furthermore, the ROC curve indicated that the prediction ability of the risk score was higher than 0.5 (AUC>0.5). The heatmap plot revealed that the expression levels of LOC646762 and the mRNAs were significantly different among the three groups of risk stratification in the 70 adult AML patients, and the results clearly indicated that most of the deceased patients who were associated with poor prognosis were in the intermediate and high risk groups (Fig. 8D).

## Discussion

The onset of AML is a complex, heterogeneous and multifactorial process. Abnormal epigenetic regulation has as an important role in the onset and progression of AML 18. However, the biological functions and the roles in risk stratification and prognosis of the majority of lncRNAs involved in epigenetic regulation have not yet been researched. To provide insights into the biological functions of the lncRNAs associated with the prognosis of adult AML patients and their role in risk stratification, we performed a comprehensive analysis of the prognostic lncRNA and mRNA RNAseq data from TCGA database in the present study. We identified the prognostic lncRNAs and their functional annotations in the modularization process. Furthermore, we analyzed the hub lncRNA and its co-expressed mRNAs in risk stratification. In particular, we validated the expression levels and risk stratification of the hub lncRNA and its co-expressed mRNAs by using patient samples. Overall, the present study revealed the co-expression networks, functional modules and risk stratification of the genes associated with prognosis in AML progression, in which lncRNAs serve an indispensable role.

A large amount of RNAseq data was obtained from the TCGA database for the prognostic analysis. First, we identified the genes associated with the prognosis of adult AML for conducting weighted co-expression network analysis in modularization. The functional modules in AML identified by GO and KEGG analysis were mainly annotated into “Fc gamma R-mediated phagocytosis”. The STRING database was used to build a PPI network, and then the network was simplified into several subnetworks based on the k-core. Subsequently, the PPI subnetworks were annotated into several functional modules by KEGG. In general, modularization contributed to analyzing the biological functions of the intricate networks of the RNAseq data in adult AML. Second, the lncRNA-LOC646762 co-expression network in the blue module attracted our attention. Several functional modules were enriched by KEGG. Subsequently, the genes associated with prognosis in the lncRNA-LOC646762 co-expression network were selected using a Kaplan-Meier curve. The hub genes were further selected from the prognostic genes by multivariate Cox regression for analysis of risk stratification. Finally, we validated the expression levels of lncRNA-LOC646762 and co-expressed CCND3, CBR1, C10orf54, CD97 and BLOC1S1 in adult AML patients and healthy volunteers by qRT-PCR, and then identified their roles in prognosis and risk stratification.

A novel lncRNA-LOC646762, located at 7p14.3, was a protective factor for adult AML patients, and overexpression of it predicted a favorable prognosis in risk stratification. In the present study, LOC646762, regulating co-expressed mRNA, may contribute to AML through the "endocytosis" signaling pathway. A previous study has demonstrated that the protein encoded by cyclin D3 (CCND3) belongs to the highly conserved cyclin family, which affects the cell cycle by regulating the transition from G(1) to the S phase, and high CCND3 expression predicts a poor outcome in patients with diffuse large B-cell lymphoma 19. The present study revealed that CCND3 is a risk factor for AML, and its overexpression predicts a poor prognosis. The protein encoded by carbonyl reductase 1 (CBR1) belongs to the short-chain dehydrogenases/reductases (SDR) family, which function as NADPH-dependent oxidoreductases 20. A previous study demonstrated that low expression of CBR1 promotes growth and proliferation of ovarian cancer 21. However, the present study indicated that overexpression of CBR1, a risk factor for AML, predicts an adverse outcome. C10orf54 is also known as V-set immunoregulatory receptor (VSIR) and its expression is significantly associated with tumor immune evasion in colorectal carcinoma tumors 22. High expression of C10orf54 is related to a poor prognosis, but C10orf54 is a protective factor when LOC646762, CCND3, CBR1, CD97 and BLOC1S1 are expressed. Overexpression of CD97, also known as adhesion G protein-coupled receptor E5 (ADGRE5), in the leukemia stem cells of AML patients is associated with a poor prognosis 23, which is in line with the results of the present study. Biogenesis of lysosomal organelles complex 1 subunit 1 (BLOC1S1) is a component of the BLOC1 multisubunit protein complex 24. High expression of BLOC1S1 predicts an adverse outcome, while BLOC1S1 overexpression is correlated with a favorable prognosis when LOC646762, CCND3, CBR1, CD97 and BLOC1S1 are expressed. In summary, risk stratification for adult AML patients can be performed based on the combination of these six prognostic genes.

In conclusion, the present study clearly demonstrates that lncRNAs serve an important role in adult AML. LncRNA-LOC646762, which may contribute to AML through the "endocytosis" signaling pathway, may act as a biomarker for predicting the survival of adult AML patients, as well as for risk stratification. However, there were many prognostic lncRNAs, including the pseudogene-derived lncRNAs and the lncRNAs that were not assigned to functional modules, that were excluded from the analysis. Furthermore, the biological functions of the lncRNAs require further identification and validation. Investigation of the additional biological characteristics of LOC646762 and their potential involvement in the disease mechanisms in a larger cohort of AML patients is necessary. This study provides a reference for a more comprehensive analysis of high-throughput data in the future.

## Supplementary Material

Table S1.

## Figures and Tables

**Table 1:** Clinical characteristics of AML patients.

Variables	Total	Testing cohort	Validation cohort	*P*-value
Number	237	167	70	
Age, mean±SD, years	51 ± 17	56 ± 16	42 ± 16	0.000
**Gender, n (%)**				0.206
Male	120 (50.6)	89 (53.3)	31 (44.3)	
Female	117 (49.4)	78 (46.7)	39 (55.7)	
**Dignosis, n (%)**				0.000
M0	15 (6.3)	15 (9.0)	0 (0.0)	
M1	39 (16.5)	36 (21.6)	3 (4.3)	
M2	54 (22.8)	37 (22.2)	17 (24.3)	
M3	27 (11.4)	16 (9.6)	11 (15.7)	
M4	40 (16.9)	35 (21.0)	5 (7.1)	
M5	53 (22.4)	21 (12.6)	32 (45.7)	
M6	4 (1.7)	2 (1.2)	2 (2.9)	
M7	3 (1.3)	3 (1.8)	0 (0.0)	
Not Classified	2 (0.8)	2 (1.2)	0 (0.0)	
**Cytogenetics, n (%)**				0.808
Normal	3 (1.3)	84 (1.8)	34 (0.0)	
Abnormal	2 (0.8)	83 (1.2)	36 (0.0)	

**Table 2:** The primers for qRT-PCR.

Target	Sequence (5' - 3')
LOC646762 (F)	CCGTAGGACTCGCAGGACTCG
LOC646762 (R)	GGTGAGAGGTGAGCTGGTAAGGAG
CCND3 (F)	GGACCTGGCTGCTGTGATTGC
CCND3 (R)	CCGTGGCGATCATGGATGGC
CBR1 (F)	CCAAGCATCCTGCGTACTGTCTG
CBR1 (R)	AAGCAGCGGCAGATTATGGACATC
C10orf54 (F)	ACCACCACTCGGAGCACAGG
C10orf54 (R)	TTGTAGACCAGGAGCAGGATGAGG
CD97 (F)	GCATTCTGTGTCTGGCTGACTCTG
CD97 (R)	CGACAGGCGGTGGCATTGAC
BLOC1S1 (F)	AAGAGGAGGCGAGAGGCTATCAC
BLOC1S1 (R)	GTTCTCCACCATTCCGATCCACTG
18S rRNA (F)	CGGCGGCTTTGGTGACTCTAGA
18S rRNA (R)	CCTGCTGCCTTCCTTGGATGTG

**Table 3:** Univariate and multivariate Cox regression analysis of LOC646762 and the co-expressed mRNAs in adult AML patients (n= 167).

Variable	Univariate analysis				Multivariate analysis		
	β	*P*- value	exp (95% CI for exp)		β	*P*- value	exp (95% CI for exp)
CCND3	0.011	0.000	1.011 (1.006-1.015)		0.011	0.008	1.011 (1.003-1.019)
CBR1	0.099	0.000	1.104 (1.065-1.144)		0.120	0.000	1.127 (1.074-1.184)
ACTR1A	0.051	0.000	1.052 (1.028-1.076)				
BLOC1S2	0.065	0.004	1.067 (1.022-1.115)				
ADCY7	0.011	0.009	1.011 (1.003-1.019)				
C10orf54	0.003	0.012	1.003 (1.001-1.005)		-0.005	0.033	0.995 (0.990-1.000)
CDH23	0.156	0.016	1.169 (1.029-1.328)				
CD97	0.004	0.000	1.004 (1.002-1.006)		0.005	0.005	1.005 (1.001-1.008)
BLOC1S1	0.015	0.035	1.015 (1.001-1.030)		-0.030	0.036	0.971 (0.944-0.998)
CD300LF	0.012	0.029	1.012 (1.001-1.023)				
ARAP1	0.014	0.003	1.014 (1.005-1.023)				
LOC646762	-0.056	0.000	0.946 (0.919-0.974)		-0.060	0.001	0.942 (0.908-0.976)
CD300A	0.018	0.001	1.018 (1.007-1.030)				
ANXA11	0.019	0.002	1.019 (1.007-1.031)				
BST2	0.006	0.003	1.006 (1.002-1.011)				
CAPZB	0.010	0.000	1.010 (1.005-1.015)				
BEST1	0.006	0.015	1.006 (1.001-1.011)				
CDK14	-0.101	0.008	0.904 (0.838-0.974)				
CDC42SE1	0.010	0.004	1.010 (1.003-1.017)				
CAP1	0.005	0.001	1.005 (1.002-1.008)				
C12orf5	0.117	0.033	1.125 (1.010-1.253)				
ARHGAP27	0.019	0.001	1.019 (1.008-1.030)				
AUH	-0.098	0.018	0.907 (0.836-0.983)				
CLTA	0.020	0.039	1.020 (1.001-1.039)				
BIN2	0.007	0.008	1.007 (1.002-1.012)				
ARHGAP30	0.012	0.006	1.012 (1.003-1.021)				
ANKRD27	-0.025	0.003	0.975 (0.959-0.991)				
ARL5B	-0.043	0.011	0.958 (0.926-0.990)				
CFL1	0.001	0.013	1.001 (1.000-1.002)				

β: regression coefficient, SE: standard error, exp (95% CI for exp): risk ratio.
